# Identification of Putative Potassium Channel Homologues in Pathogenic Protozoa

**DOI:** 10.1371/journal.pone.0032264

**Published:** 2012-02-21

**Authors:** David L. Prole, Neil V. Marrion

**Affiliations:** 1 Department of Pharmacology, University of Cambridge, Cambridge, United Kingdom; 2 School of Physiology and Pharmacology, University of Bristol, Bristol, United Kingdom; Institut national de la santé et de la recherche médicale - Institut Cochin, France

## Abstract

K^+^ channels play a vital homeostatic role in cells and abnormal activity of these channels can dramatically alter cell function and survival, suggesting that they might be attractive drug targets in pathogenic organisms. Pathogenic protozoa lead to diseases such as malaria, leishmaniasis, trypanosomiasis and dysentery that are responsible for millions of deaths each year worldwide. The genomes of many protozoan parasites have recently been sequenced, allowing rational design of targeted therapies. We analyzed the genomes of pathogenic protozoa and show the existence within them of genes encoding putative homologues of K^+^ channels. These protozoan K^+^ channel homologues represent novel targets for anti-parasitic drugs. Differences in the sequences and diversity of human and parasite proteins may allow pathogen-specific targeting of these K^+^ channel homologues.

## Introduction

Protozoan parasites are major contributors to worldwide disease [1]. They include apicomplexan parasites such as *Plasmodium* spp. (malaria), *Toxoplasma gondii* (toxoplasmosis), *Cryptosporidium* spp. (cryptosporidiosis, diarrhoea) and *Babesia bovis* (babesiosis), as well as the kinetoplastid parasites *Trypanosoma* spp. (sleeping sickness, Chagas' disease) and *Leishmania* spp. (leishmaniasis). These parasites are together responsible for billions of infections and hundreds of thousands of deaths each year [1], [2]. Other protozoan parasites causing widespread disease include *Giardia intestinalis* (giardiasis), *Entamoeba histolytica* (dysentery) and *Trichomonas vaginalis* (trichomoniasis). Current treatments for diseases caused by protozoa are often ineffective or poorly tolerated, and emergence of drug resistance is an imminent threat to their efficacy [3]–[5]. New therapeutic targets and drugs are therefore needed.

K^+^ channels are a diverse family of transmembrane proteins, which form K^+^-selective pores and mediate K^+^ flux across membranes [6], [7]. K^+^ channels are essential components in a multitude of homeostatic and signalling pathways and are present in animal cells [6], plants [8], [9], fungi [10], [11] and many bacteria [7], [12]. Only a handful of organisms appear to lack K^+^ channels completely, and most of these are bacteria that are obligate parasites [7], [12]. Many K^+^ channels are present in free-living protozoa such as *Paramecium* [13], but little is known about the existence and physiological role of K^+^ channels in pathogenic protozoa, many of which spend part of their life cycles as intracellular parasites. K^+^ channels are known to exist in *Plasmodium* spp. [14]–[16] and K^+^-conductive pathways have also been observed in *Trypanosoma cruzi* [17], but the molecular identity of the channels underlying these latter K^+^ fluxes is unknown.

**Figure 1:**
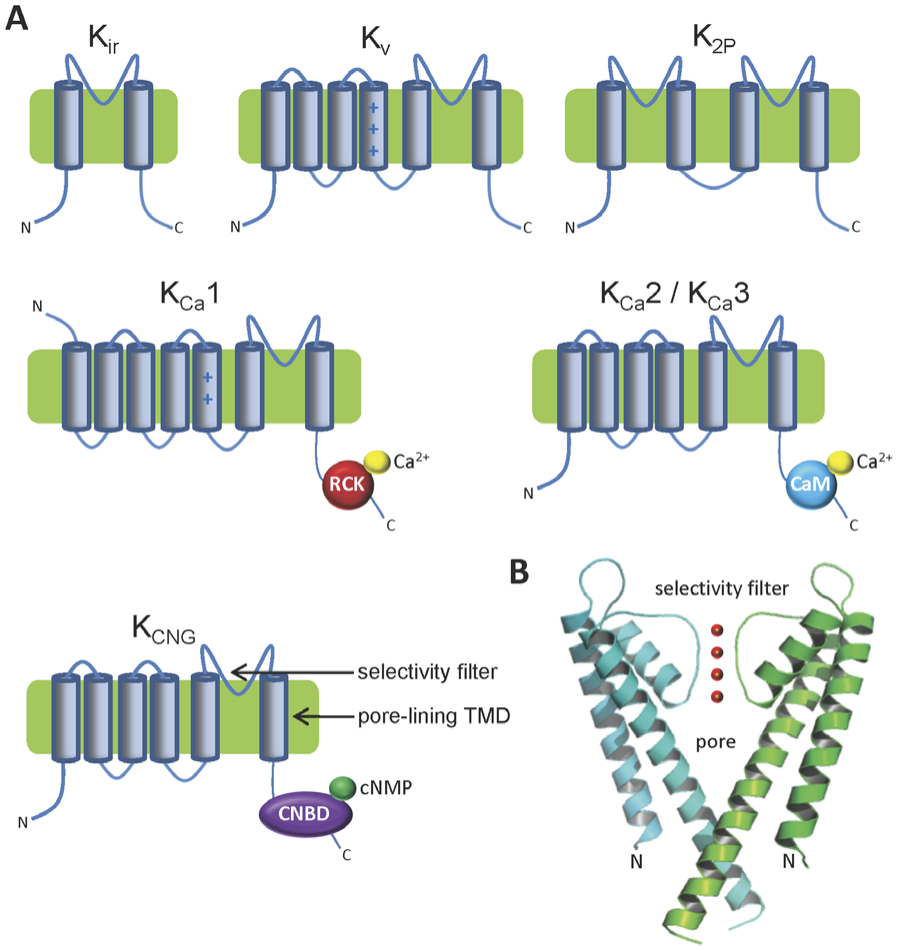
K^+^ channel families. (**A**) Topology diagrams of K^+^ channel subunits, showing locations of transmembrane domains (TMDs), functional domains and termini. Plus signs denote charged basic residues within the voltage sensor (S4) region of K_v_ and K_Ca_1 channels. In contrast to other K^+^ channel subunits, K_Ca_1.1 channel subunits have extracellular N-termini [145], [146]. RCK denotes a Ca^2+^-binding regulator of conductance of K^+^ channels domain, which also binds a variety of other ionic ligands in different channels [60]–[63]. CaM denotes calmodulin (CaM) bound to a CaM-binding site within the channel subunit [60], [64]. CNBD denotes a cyclic nucleotide monophosphate (cNMP) binding site [65]; (**B**) A crystal structure of the KcsA pore domain is shown (PDB accession number 1K4C) [147], with only the TMDs and pore loops of two subunits depicted for clarity. Red circles represent a number of the K^+^ ions in the selectivity filter.

Several subtypes of K^+^ channel exist, including voltage-gated (K_v_), inward rectifier (K_ir_), two-pore (K_2P_), calcium-gated (K_Ca_) and cyclic nucleotide-gated (K_CNG_) channels [6] (**Figure 1A**). These channels are all formed by a tetrameric arrangement of pore-forming domains, contributed to by each of four monomeric subunits in most channels, except K_2P_ channels which exist as a dimer of subunits, with each subunit containing two domains that contribute to the pore (**Figure 1A**) [6], [18]. The K^+^-conducting pore region is comprised of a tetrameric arrangement of re-entrant pore loops (P-loops), part of which forms the selectivity filter, together with the following pore-lining transmembrane domains (TMDs) from each subunit, which form the inner pore (**Figures 1A and 1B**). Diversity of K^+^ channels is increased by subunit heteromerization and by the association of auxiliary subunits, such as K_v_β, KCNE, KChIP, BKβ, and sulfonylurea (SUR) subunits, which alter the functional properties, trafficking, modulation and pharmacology of K^+^ channels [19]–[22].

In many cell types K^+^ channels are found mainly in the plasma membrane, but they are also found in the membranes of intracellular organelles such as mitochondria [23], [24], nuclei [25]–[28], endosomes [29], endoplasmic reticulum [30], secretory vesicles [31], [32] and intracellular vacuoles [33]–[35]. Physiological roles of K^+^ flux include setting or altering membrane potentials, effecting osmolyte homeostasis, altering enzyme activity, promoting mitogenesis or apoptosis, and facilitating transmembrane transport processes [6]–[9], [12], [36]. Pharmacological or genetic perturbation of K^+^ channel activity has profound effects on cell function in many organisms, suggesting that parasite homologues of these channels might represent novel drug targets. Consistent with this, disruption of K^+^ channel function in *Plasmodium falciparum* and *Plasmodium berghei* is lethal to these parasites [16], [37].

Recent advances in genomics have resulted in whole-genome sequencing of many pathogenic protozoa [1], [38]–[56]. In this study we examine the genomes of pathogenic protozoa comprehensively, using diverse K^+^ channel sequences from mammals, plants, fungi, bacteria and archaea, to search for the presence of predicted proteins that may fulfil roles as K^+^ channels. We show that genes encoding homologues of K^+^ channels exist in all pathogenic protozoa examined. Sequence divergence of putative protozoan channels from their human counterparts in regions that are known to be important for channel activation, ion conduction or drug binding may result in distinct pharmacological profiles. These parasite channels may therefore represent novel targets for anti-parasitic therapy.

## Results

### Identification and classification of K^+^ channel homologues

**Figure 2:**
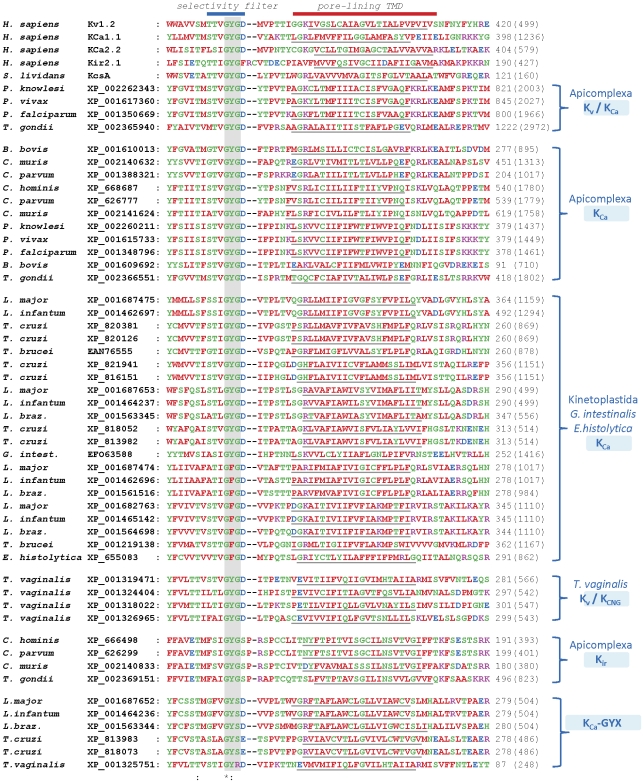
Multiple sequence alignment of protozoan K^+^ channel homologues with the pores of mammalian K^+^ channels. Predicted pore-lining TMD regions are underlined. The GXG motif of human K^+^ channels is shaded in grey. Total number of residues in each protein is indicated in parentheses to the right of each sequence. *L. braz*. denotes *L. braziliensis*, and *G.intest*. denotes *G. intestinalis*. The proteins XP_001609692 and XP_001350669 encoded by the *P. falciparum* genome are identical to the previously described PfKch2 and PfKch1 proteins respectively [14], [16]. The proteins XP_001610013 and XP_668687 are identical to previously identified K^+^ channel homologues in *B. bovis* and C*ryptosporidium hominis* respectively [16]. The proteins labelled GYX have GYRD, GYSD or GYSE-containing selectivity filter regions, suggesting a lack of K^+^ selectivity or function.

The defining feature of K^+^ channels is their selectivity for K^+^ ions, which is conferred by residues within the selectivity filter region of the pore [57] (**Figure 1**). Diverse mammalian K^+^ channels show sequence similarity in the selectivity filter region, with a core selectivity filter motif of XXGXGX, most commonly TXGYGD [58]. K^+^ selectivity is known to be tolerant of some sequence variation in this selectivity filter motif [59] as well as in the outer and inner pore regions, and such variation exists between channel subtypes [58]. For example, selectivity filter sequences of K^+^-selective channels include TIGYGF (*eg.* K_ir_2.1, K_ir_2.3), TIGYGL (*eg.* K_ir_2.2), XXGFGX (*eg.* K_ir_6.2, ERG, EAG, mouse K_Ca_1.1), and XXGLGD (*eg.* some K_2P_) [58]. We therefore searched parasite genomes using diverse K^+^ channel sequences from humans, plants, fungi, bacteria and archaea (see **Methods**), which together cover most known K^+^-selective pore sequences. We identified predicted protein products in the genomes of pathogenic protozoa, which display significant sequence similarity to K^+^ channels in the pore region, including the selectivity filter (**Table 1** and **Figure 2**). These proteins also satisfy other criteria for defining them as putative K^+^ channel homologues, such as the presence of multiple TMDs (see **Methods**). These homologues may therefore function as K^+^-selective channels in protozoan parasites. Homologues were classified according to the family of human K^+^ channel to which they showed greatest sequence similarity, and according to the presence of conserved functional domains (**Figure 1A**) such as putative voltage sensors, Ca^2+^-sensing regulator of conductance (RCK) domains of K_Ca_ channels [60]–[63], calmodulin (CaM)-binding domains (CaMBDs) [60], [64], or cyclic nucleotide-binding domains (CNBDs) [65] (**Table 1** and **Figure 2**). The *P. falciparum* proteins XP_001609692 and XP_001350669 are identical to the previously described PfKch2 and PfKch1 proteins respectively (also known as PfK2 and PfK1 respectively) [14], [16]. The homologues in *B. bovis* (XP_001610013) and *Cryptosporidium hominis* (XP_668687) have been previously identified by similarity to a K^+^ channel homologue in *P. berghei* [16].

**Table 1:** Identity of K^+^ channel homologues in pathogenic protozoa.

Parasite	K_v_ or K_CNG_ *	K_Ca_	K_ir_
*Plasmodium falciparum*	XP_001350669 (12) (K_v_)	XP_001348796 (10)	NF
		XP_001350669 (12)	
*Plasmodium knowlesi*	XP_002262343 (13) (K_v_)	XP_002260211 (8)	NF
		XP_002262343 (13)	
*Plasmodium vivax*	XP_001617360 (10) (K_v_)	XP_001615733 (8)	NF
		XP_001617360 (10)	
*Toxoplasma gondii*	XP_002365940 (8) (K_v_)	XP_002366551 (10)	XP_002369151 (4)
		XP_002365940 (8)	
*Cryptosporidium hominis*	NF	XP_668687 (8)	XP_666498 (2)
*Cryptosporidium muris*	XP_002140632(10)(K_v_)	XP_002140632 (10)	XP_002140833 (2)
		XP_002141624 (11)	
*Cryptosporidium parvum*	NF	XP_626777 (8)	XP_626299 (2)
		XP_001388321 (6)^b^(K_Ca_4)	
*Babesia bovis*	NF	XP_001609692 (2)	NF
		XP_001610013 (5)	
*Giardia intestinalis*	NF	EFO63588 (9)	NF
*Entamoeba histolytica*	NF	XP_655083 (6)	NF
*Leishmania major*	NF	XP_001687475 (8)	NF
		XP_001687474 (6)	
		XP_001682763 (7)	
		XP_001687653 (6)^b^(K_Ca_2/K_Ca_3)	
		XP_001687652^a^	
*Leishmania infantum*	NF	XP_001462697 (7)	NF
		XP_001462696 (6)	
		XP_001465142 (9)	
		XP_001464237 (6)^b^(K_Ca_3/K_Ca_2)	
		XP_001464236^a^	
*Leishmania braziliensis*	NF	XP_001561516 (6)	NF
		XP_001564698 (6)	
		XP_001563345 (7)^b^(K_Ca_3/K_Ca_2)	
		XP_001563344^a^	
*Trypanosoma brucei*	NF	EAN76555 (7)	NF
		XP_001219138 (6)	
*Trypanosoma cruzi*	NF	XP_821941 (8)	NF
		XP_816151 (8)	
		XP_820381 (6)	
		XP_820126 (6)	
		XP_818052 (8)^b^(K_Ca_3/K_Ca_2)	
		XP_813982 (8)^b^(K_Ca_3/K_Ca_2)	
		XP_813983^a^	
		XP_818073^a^	
*Trichomonas vaginalis*	XP_001324404 (6)*	NF	NF
	XP_001319471 (6)*		
	XP_001326965 (6)*		
	XP_001318022 (6)*		
	XP_001325751 (2)* a		

Protein accession numbers are shown and NF denotes that no homologues were found. Number of predicted TMDs is indicated in parentheses. K^+^ channel homologues are classified on the basis of closest similarity to a particular subtype of human K^+^ channel subunit and according to the presence of characteristic functional domains, such as a charged TMD4 (K_v_), the presence of a cyclic nucleotide-binding domain CNBD (K_CNG_), or the presence of RCK domains (K_Ca_). Where a protein showed similarity to more than one class of K^+^ channel, its accession number is shown in both relevant columns (*eg.* all K_v_ homologues are also K_Ca_ homologues).

* K_CNG_ homologues which contain a CNBD, but also a charged TMD4;

a likely non-selective or non-functional due to GYRD, GYSD or GYSE selectivity filter motifs;

b no putative RCK domains or calmodulin-binding domains (CaMBDs) were detected in these proteins by searching the Conserved Domains Database, but in BLASTP searches of the human genome these proteins showed greatest sequence similarity to the K_Ca_ channel subtypes indicated in parentheses. The *P. falciparum* proteins XP_001609692 and XP_001350669 are identical to the previously described PfKch2 and PfKch1 proteins respectively [14], [16]. The *B. bovis* (XP_001610013) and *C. hominis* (XP_668687) proteins have been identified previously as orthologues of PfKch1 in *P. berghei* [16]. In addition to the K^+^ channel homologues shown, homologues of putative adenylyl cyclase/K^+^ channel fusion proteins [123], [124] that contain GXG motifs after their TMD6 domains were also identified in *P. falciparum* (XP_001348216), *P. knowlesi* (XP_002260946), *P. vivax* (XP_001616904), *T. gondii* (XP_002368352, XP_002370938 and XP_002367966), *C. muris* (XP_002140763), *C. hominis* (XP_666311) and *C. parvum* (XP_626352). Homologues of these proteins were absent in all other parasites examined.

### Putative pore regions of K^+^ channel homologues

Parasite K^+^ channel homologues have a variety of putative selectivity filter sequences, including XXGYGD, XXGFGD and XXGYGS (henceforth referred to as GYGD, GFGD and GYGS) (**Figure 2**). The sequence GYGD is common in mammalian K^+^ channels [58], while GFGD sequences also occur in several channels, including human K_ir_, ERG and EAG, as well as mouse K_Ca_1.1 [58]. In contrast, GYGS is very rare in mammals [58], but occurs in several of the parasite K^+^ channel homologues examined, which are most closely related to human K_ir_ channels (**Figure 2**). The identity of the residue immediately following the second glycine of the selectivity filter motif affects conductance and rectification as well as gating, binding of K^+^ and potency of inhibitors [66], [67]. An aspartate residue at this locus reduces block by Cd^2+^ [68] and tetraethylammonium (TEA) [66], suggesting that sensitivity to these and other blockers may differ between GYGD-containing human K^+^ channels and the GYGS-containing protozoan K^+^ channel homologues. These GYGS-containing parasite channels also contain a glutamate residue at an analogous position to the conserved P-loop glutamate residue of K_ir_ channels and KcsA (E138 in K_ir_2.1, **Figure 2**) that is involved in gating at the selectivity filter [69], [70]. In contrast, these homologues lack an aspartate residue (D172 in K_ir_2.1) within the inner helix that confers strong inward rectification on some mammalian K_ir_ channels [71]. These parasite GYGS-containing K^+^ channel homologues may therefore show rectification and sensitivity to extracellular blockers that differs from human K_ir_ channels, but may exhibit similarities in gating.

A separate group of proteins (henceforth termed GYX-containing proteins) was also defined in kinetoplastid *Leishmania* and *Trypanosoma* parasites as well as *T. vaginalis*. These proteins have GYSD/E or TIGYRD sequences respectively at the putative selectivity filter locus, but otherwise show pronounced similarity to various mammalian K^+^ channel pores (**Figure 2** and **Table 1**). Each of the genes encoding these GYX proteins in *Leishmania* spp. and *T. cruzi* is present in a chromosomal region that is closely adjacent to genes encoding one of the other K^+^ channel homologues identified, which have canonical GYG- or GFG-containing selectivity filters. This suggests that these GYX proteins may be paralogues that arose via gene duplication. Mutations of the second glycine in the GXG motif have been shown to eliminate K^+^ selectivity and function in K^+^ channels [72], suggesting that these GYX proteins may be non-selective cation channels, or may be non-functional.

The selectivity filter and adjoining P-loop form the binding site for many drugs and toxins that block K^+^ channels [73]–[78]. Likewise, the inner pore region of K^+^ channels often binds a variety of drugs [77], [79]–[84]. Sequence differences between human and parasite homologues in these regions (**Figure 2**) suggest that it may be possible to isolate drugs that bind specifically to the inner pore of parasite proteins. For example, many protozoan K_Ca_ homologues differ from human K_Ca_1.1 at a locus (equivalent to G376 of hK_Ca_1.1 in **Figure 2**) that determines sensitivity to the potent K_Ca_1.1-specific fungal toxin paxilline [85]. This suggests that human K_Ca_ channels and protozoan K_Ca_ channel homologues might exhibit different sensitivity to paxilline and related drugs.

### K_v_ channel homologues

**Figure 3:**
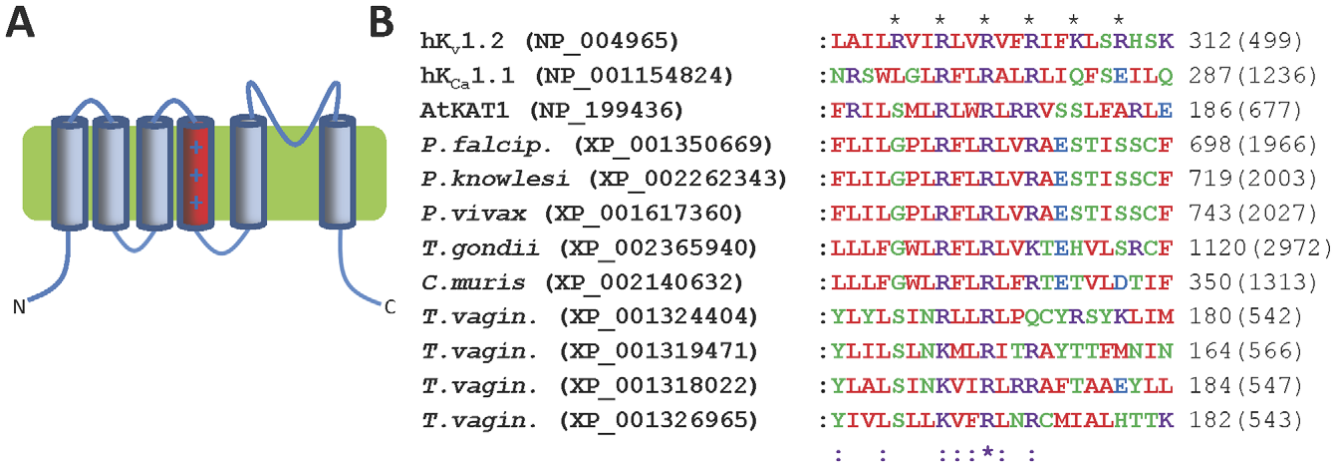
Protozoan K^+^ channels containing charged TMD4 regions. (**A**) Topology diagram of K_v_1.2, with the positively charged TMD4 shown in red; (**B**) Multiple sequence alignment of the TMD4 regions of human voltage-gated K_v_1.2 and K_Ca_1.1, plant voltage-gated KAT1 and the predicted TMD4 regions of those protozoan K^+^ channel homologues containing at least three basic residues within this region. Asterisks above the alignment indicate basic residues involved in voltage sensing in K_v_1.2 channels.

The genomes of *Plasmodium* spp., *T. gondii*, *Cryptosporidium muris* and *T. vaginalis* contain genes encoding homologues of K_v_ channels, with TMD4 regions that contain a number of regularly spaced basic residues, similar to the S4 voltage sensor sequences of K_v_ channels [60] (**Table 1**, **Figure 1** and **Figure 3**). The apicomplexan K_v_ homologues also show sequence similarity to the C-terminal tails of K_Ca_1 channels, including the RCK domains that bind Ca^2+^ or other ions [61], [62], [86] (*data not shown*). This suggests that these parasite homologues may be dually modulated by voltage and ions such as Ca^2+^, Mg^2+^ or H^+^. In contrast, K_v_ homologues in *T. vaginalis* all contain conserved CNBDs in their C-terminal tails (*see later*), suggesting that they, like mammalian hyperpolarization-activated cyclic nucleotide-gated non-selective (HCN) channels [65], may be dually modulated by voltage and cyclic nucleotides such as cyclic adenosine monophosphate (cAMP) or cyclic guanosine monophosphate (cGMP).

### K_Ca_ channel homologues

The most common genes encoding K^+^ channel homologues in protozoan parasites are those encoding K_Ca_ channel homologues (**Table 1** and **Figure 2**). This is consistent with the presence of complex Ca^2+^-signalling machinery in protozoa [2], [87], [88]. Known K_Ca_ channels form two main families. Proteins in one family are activated by Ca^2+^ (and in some cases by other ions such as Mg^2+^, H^+^ and Na^+^) via direct binding to domains within the channel, including the C-terminal RCK domains of K_Ca_1 channels [60]–[63] (**Figure 1A**). Proteins in the second family, which includes K_Ca_2 and K_Ca_3 channels, are activated by Ca^2+^ via binding of their C-terminal tails to the accessory Ca^2+^-binding protein CaM [60], [64] (**Figure 1A**). Many of the protozoan K_Ca_ channel homologues show sequence similarity to the C-terminal tails of K_Ca_1 or K_Ca_2/3 channels, including the RCK domains and CaMBDs (*data not shown*), suggesting that their activity may be regulated by Ca^2+^, or by other ions such as Mg^2+^, H^+^ or Na^+^.

**Figure 4:**
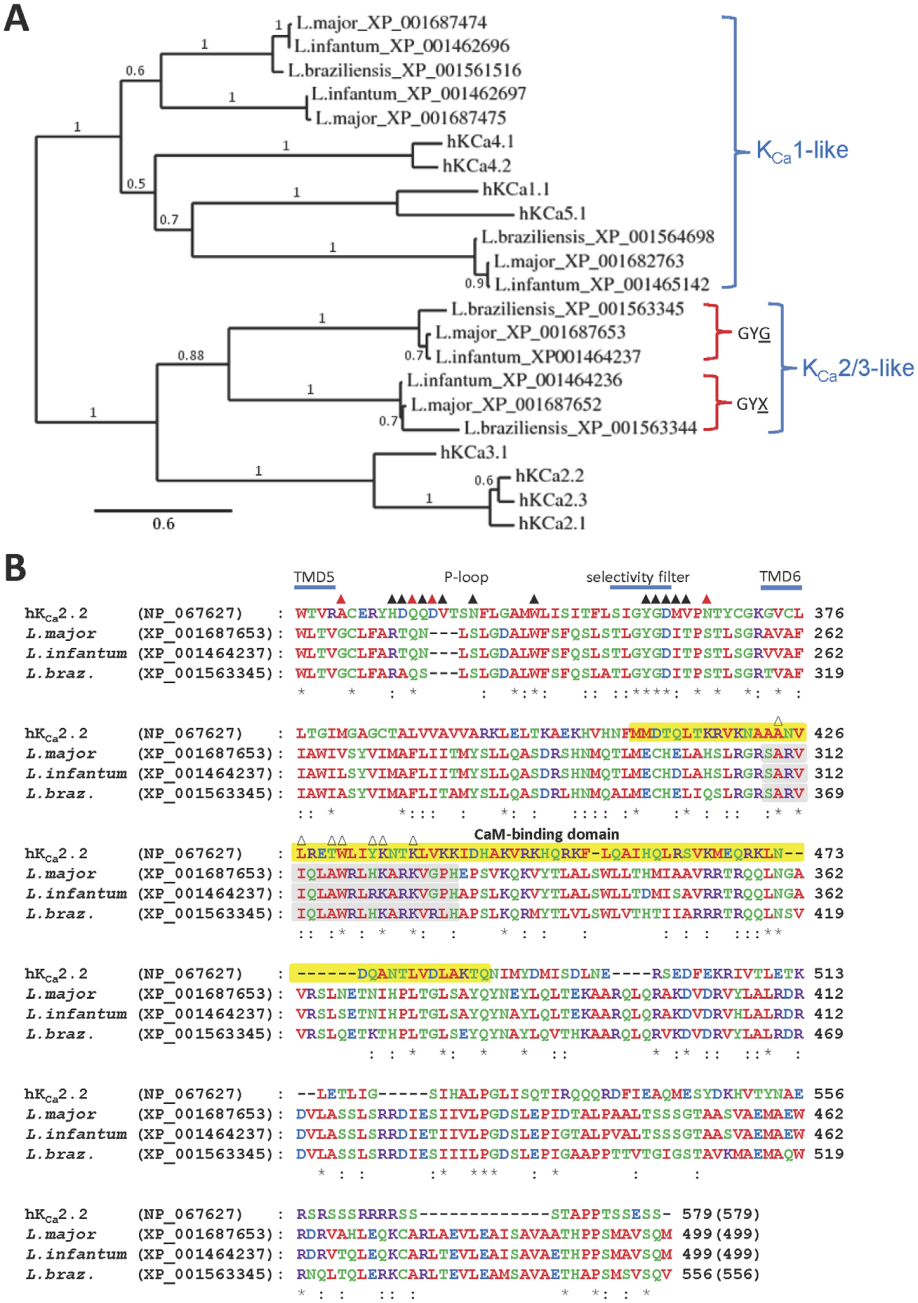
K_Ca_ channel homologues in *Leishmania* parasites. (**A**) Phylogram showing the relationship between the sequences of human K_Ca_ channels and K^+^ channel homologues in *Leishmania* spp. (*see Methods*). Branch length scale bar and branch support values are shown (*see Methods*). Two main groups of *Leishmania* proteins (K_Ca_1-like and K_Ca_2/3-like) are indicated. Selectivity filter GYG-containing K_Ca_2/3-like channels and their GYX-containing putative paralogues are also indicated; (**B**) Multiple sequence alignment of human K_Ca_2.2 (small-conductance Ca^2+^-activated SK2 channels) with the GYG-containing K_Ca_2/3-like homologues in *Leishmania* spp. Selectivity filter, TMD and P-loop regions are indicated above the alignment. Filled triangles above the alignment indicate K_Ca_2.2 residues implicated in binding of inhibitory toxins. Those previously shown experimentally to alter toxin effects are indicated by red triangles, while additional residues implicated via molecular modelling are indicated by black triangles [78]. The yellow shaded region denotes the fragment of K_Ca_2.2 that binds CaM [64] and open triangles indicate specific K_Ca_2.2 residues known to be involved in binding CaM.

We were most interested in the multiple K_Ca_ channel homologues present in *Leishmania* parasites and chose to interrogate these homologues further (**Figure 4**). Based on full-length alignments and phylogenetic relationships (**Figure 4A**), K_Ca_ homologues in *Leishmania* parasites could be grouped into K_Ca_1-like proteins (which showed similarity to the discontinuous RCK domains of hK_Ca_1.1, *data not shown*) and K_Ca_2/3-like proteins which had greater similarity to K_Ca_2/3 channels. The latter group contain homologues with canonical GYG-containing putative selectivity filter regions, as well as homologues with GYX-containing selectivity filters (**Figure 4A** and **Figure 2**). These K_Ca_2/3-like proteins have consensus IQ-motifs in their C-terminal tails, shortly after the final TMD, which are predicted to form CaMBDs (shown in **Figure 4B** for the GYG-containing homologues). These predicted CaMBDs overlap closely with the well-characterized CaMBD of K_Ca_2.2 (**Figure 4B**) and share sequence identity at several loci that are critical for binding of CaM to human K_Ca_2.2 (**Figure 4B**, open triangles) [64]. This suggests that these parasite homologues may form K_Ca_ channels whose activity is controlled by Ca^2+^ acting via bound CaM, similar to human K_Ca_2 channels. The K_Ca_ homologues in *Leishmania* (as well as the other protozoa examined) show considerable sequence divergence from human K_Ca_2 channels in the P-loop and selectivity filter, including at positions implicated in the binding of isoform-specific drugs and toxins to K_Ca_2 channels (filled triangles in **Figure 4B**) [78]. These homologues also differ markedly from human K_Ca_2 channels in the predicted TMD3-TMD4 linker region (*data not shown*), which is also an important determinant of drug binding in K_Ca_2 channels [89]. This suggests that these parasite K_Ca_ homologues may exhibit unique pharmacological profiles.

#### K_CNG_ homologues

**Figure 5:**
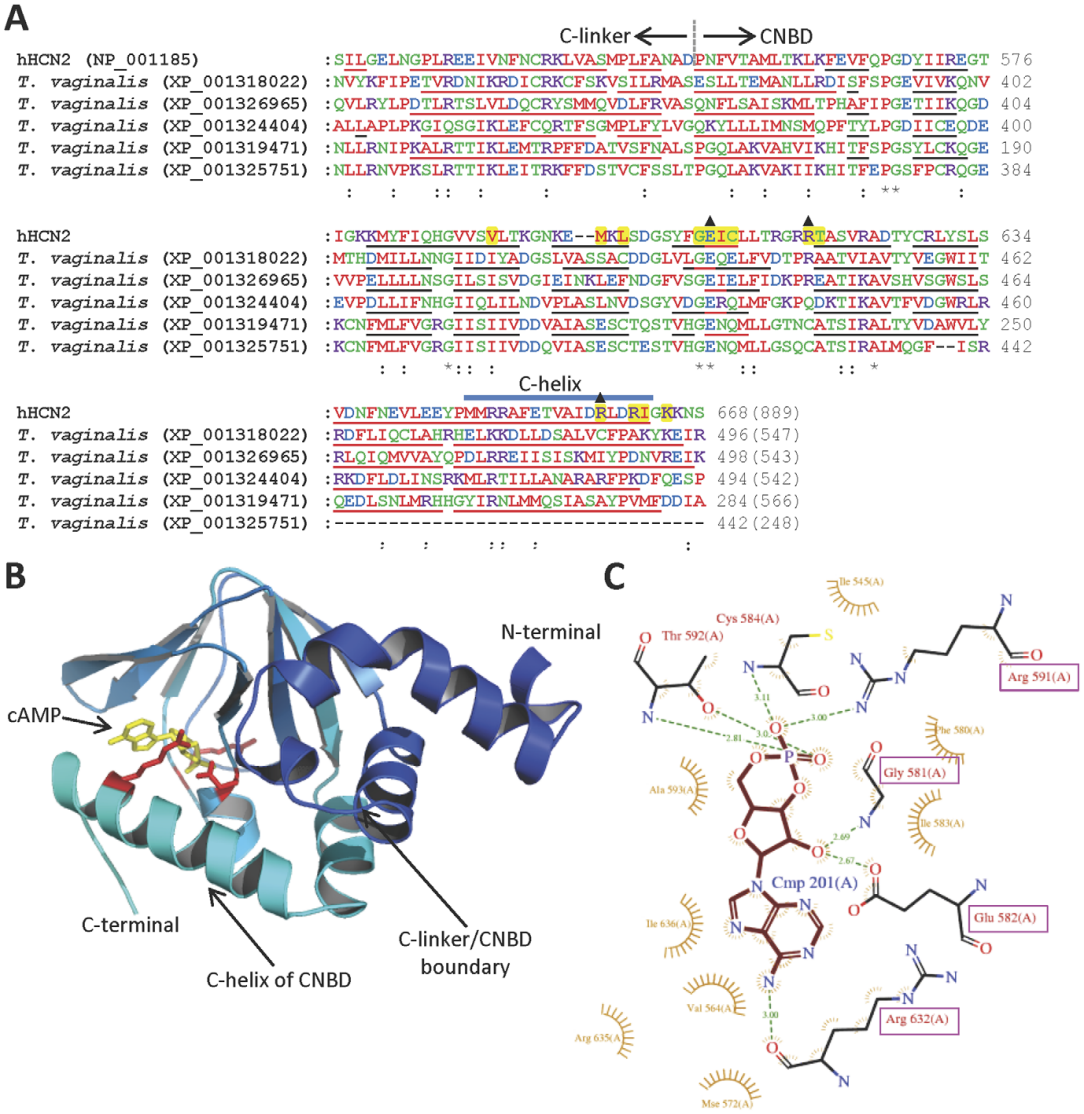
K^+^ channel homologues in *T. vaginalis* contain domains similar to mammalian cyclic nucleotide-binding domains. (**A**) Multiple sequence alignment of the C-terminal CNBD of human HCN2 (residues 516–668) with the putative CNBD-containing regions of protozoan K_CNG_ homologues. The boundary between the C-linker and CNBD of HCN2, as well as the C-helix of the CNBD [65], are indicated. Residues of HCN2 that are shaded in yellow are those known to be directly involved in binding cNMP [65], [90], [148]. Asterisks below the alignment indicate absolutely conserved residues, while colons indicate conservation of physicochemical properties (ClustalW2). Predicted secondary structure was determined using SABLE (http://sable.cchmc.org) [144] and indicated by red underline (predicted alpha helical) or black underline (predicted beta-sheet). (**B**) Crystal structure of the CNBD of mouse HCN2 in complex with cAMP (a fragment of PDB accession number 1Q5O) [65]. Only the region encompassing the residues analogous to those of hHCN2 in the alignment in **Figure 5A** are shown (residues 490–641 of mHCN2, equivalent to residues 516–668 of hHCN2). Bound cAMP is shown in yellow, and side-chains of some key residues important for cAMP binding [90] are shown in red (E582, R591 and R632 of mouse HCN2, equivalent to E609, R618 and R659 respectively of hHCN2 – labelled with filled triangles in **Figure 5A**); (**C**) A representation of the coordination of cAMP by specific residues within the CNBD of mHCN2, made using LIGPLOT v4.5.3 [149]. Labels of mHCN2 residues interacting with cAMP that are conserved in parasite K_CNG_ homologues are shown in magenta boxes.

Several genes encoding K^+^ channel homologues with conserved CNBDs (identified by searches of the Conserved Domains Database, NCBI) are present in *T. vaginalis*, but are absent from all other protozoa examined (**Table 1** and **Figure 5**). These putative K^+^ channel homologues exhibit sequence similarity to the CNBD of the well-characterized HCN2 channel that is activated by cAMP and cGMP, including some conserved residues involved in binding of cyclic nucleotides [65] (**Figures 5A, 5B and 5C**). Predicted secondary structure of the CNBD domain in HCN2 (**Figure 5A**) closely matched secondary structure in the crystal structure of HCN2 [65] (**Figure 5B**), suggesting that secondary structure of the parasite proteins could also be predicted accurately. Predicted secondary structure of the putative CNBDs of protozoan K_CNG_ channel homologues in most cases closely matched that of HCN2 (**Figures 5A and 5B**), suggesting that the structure of these domains is also conserved in human HCN2 and *T. vaginalis* K_CNG_ homologues. One exception was the C-terminal end of this domain in the GYX protein XP_001325751 (which as discussed earlier may not form functional or K^+^-selective channels), which lacked entirely the final alpha-helical stretch of HCN2 termed the C-helix, which is involved in binding and efficacy of cyclic nucleotides in HCN2 [90] (**Figure 5A** and **Figure 5B**). Hence most of the protozoan K_CNG_ homologues identified here may contain conserved CNBDs that bind cyclic nucleotides. The cyclic nucleotide selectivity of binding and efficacy in these putative channels cannot at present be predicted on the basis of sequence alone, and will require experimental testing. Unlike the non-selective mammalian HCN and CNG channels, but similar to prokaryotic K_CNG_ channels [7], these *T. vaginalis* proteins contain canonical K^+^ channel selectivity filter motifs (T/AXGYGD), suggesting that they may be K^+^-selective. We also searched parasite genomes with sequences of the K^+^-permeable (but non-selective) mammalian cyclic nucleotide-gated non-selective cation (CNG) channels and hyperpolarization-activated cyclic nucleotide-gated non-selective cation (HCN) channels, but no additional homologues were found.

### K_ir_ channel homologues

Among the protozoan genomes examined, genes encoding K_ir_ channel homologues are found only in the genomes of *Cryptosporidium* spp. and *T. gondii* (**Table 1** and **Figure 2**). K_ir_ channels are widespread among many organisms, and many subtypes exist that are differentially regulated by diverse stimuli including adenosine triphosphate (ATP), G-protein activation, phospholipids, and divalent cations [71]. As discussed earlier, the predicted pore region of the protozoan K_ir_ channel homologues shows some differences to human K_ir_ channels, which may confer unique characteristics on these homologues. The K_ir_ homologue in *T. gondii* shows most similarity to human ATP-modulated K_ir_6.2 and G-protein activated K_ir_3 channels (*data not shown*), suggesting that cytosolic ATP or G-proteins within parasites may regulate this homologue. K_ir_ homologues in *Cryptosporidium* spp. were also most similar to human ATP-modulated K_ir_6, as well as K_ir_2 channels (*data not shown*), suggesting that these homologues might also be regulated by cytosolic ATP within parasites.

### K_2P_ channel homologues

We used the sequences of human and yeast K_2P_ channels to search for predicted proteins with both sequence similarity to K_2P_ channels [18], [91] and at least four predicted TMDs in two distinct regions, each with credible potential as selectivity filter regions. Using these criteria, we found no evidence for genes encoding homologues of K_2P_ channels in the parasite genomes examined. Although the K_ir_ homologue in *T. gondii* contained four predicted TMDs in two regions, only the second pair of TMDs had an intervening sequence with similarity to canonical selectivity filters (*data not shown*).

### Protozoan parasites lack homologues of K^+^ channel auxiliary subunits

Many K^+^ channels are associated with auxiliary proteins that can change their biophysical properties, localization or regulation by cellular signalling pathways [19], [20]. These include the KCNE, KChIP and K_v_β subunits that alter K_v_ channel activity and localization [20], [21]. Other auxiliary subunits include the BKβ family of subunits that alter BK channel activity and pharmacology [20], and the sulfonylurea (SUR) subunits that are responsible for the nucleotide-diphosphate sensitivity and pharmacological profile of K_ir_6 channel complexes [92], [93]. We searched the genomes of protozoan pathogens for genes encoding predicted proteins with similarity to these auxiliary subunits, but none were found. This suggests that the protozoan parasites examined here lack conventional auxiliary subunits of K^+^ channels.

## Discussion

All protozoan genomes examined contain genes encoding K^+^ channel homologues (**Table 1**), suggesting that these putative channels have widespread and conserved physiological functions in these organisms. Many of these putative K^+^ channels are not yet annotated in available pathogen databases (http://eupathdb.org/eupathdb) [94]. Genes encoding homologues of most of the major families of K^+^ channel (K_v_, K_Ca_, K_ir_ and K_CNG_ channels) are present in protozoan genomes, although genes encoding homologues of K_2P_ channels appear to be absent. Experimental studies will be required to confirm the expression and function in parasites of these putative K^+^ channel homologues.

In mammalian cells, most plasma membrane K^+^ channels mediate K^+^ efflux, due to the common occurrence of large transmembrane K^+^ concentration gradients and relatively depolarized membrane potentials. Outward rectification or depolarization-induced activation of the channels themselves also contributes to selective efflux of K^+^ in many cases. In contrast, some plasma membrane K^+^ channels are capable of mediating K^+^ influx [95], [96], due to unusual K^+^ concentration gradients or membrane potentials. Inward rectification [71] and hyperpolarization-induced activation [97]–[99] also facilitate selective K^+^ influx in some cases. Protozoan parasites in many cases spend part of their lifecycle within cells and part in an extracellular environment. The K^+^ concentrations of these environments in a mammalian host differ by ∼40-fold (4 mM extracellular K^+^ versus 155 mM intracellular K^+^) [100]. Depending on the lifecycle stage, the concentration gradient for K^+^ flux across the parasite plasma membrane may therefore differ considerably. In this context it is interesting that *Plasmodium* parasites induce alterations of the Na^+^/K^+^ ratio in host cells and thereby reduce intracellular K^+^ concentration [101]–[104]. Whether other parasites also exert similar effects on host cells is unknown. Whether influx of K^+^ occurs through K^+^ channel homologues in the plasma membrane of intracellularly located parasites, and whether this might be exploited therapeutically (*see later*) remains to be explored.

Protozoan K_v_ channel homologues may be regulated by transmembrane voltage. Most K_v_ channels are activated by depolarization [60], while a few are activated by hyperpolarization [98], [99]. However, known depolarization-activated and hyperpolarization-activated channels show similar voltage sensor sequences [105], making it difficult to determine the polarity of voltage dependence on the basis of sequence alone. Further experimental studies will therefore be required to define the properties of these homologues. The vast majority of mammalian K_v_ channels are present and functional in the plasma membrane, which experiences the most substantial changes in transmembrane potential. Parasite K_v_ channel homologues therefore seem likely to reside within the plasma membrane of these organisms, although this will require experimental testing. Parasite K^+^ channels may also be expressed in the membranes of their host cells. For example, in the case of *P. falciparum*, the PfKch1 channel is located in the plasma membrane of the host erythrocyte, while PfKch2 is mainly located in the parasite [15]. The presence of putative K_v_ channel homologues in protozoa suggests that these organisms may experience dynamic physiological changes in membrane potential. The plasma membrane potentials of some protozoa have been estimated. For example, the intraerythrocytic form of *P. falciparum* has an estimated membrane potential of −95 mV, which is contributed to by K^+^ flux [106]. Similarly, the plasma membrane potential of the bloodstream form of *T. brucei* has been measured as −82 mV and is mainly due to K^+^ flux [107]. In addition, the membrane potential of *Leishmania donovani* amastigotes has been measured as between −90 and −113 mV and is also contributed to by K^+^ flux [108]. However, whether the membrane potentials of protozoan parasites change between stages of the lifecycle or in response to environmental stimuli, and whether the K_v_ channel homologues identified here respond to such changes is unknown.

Genes encoding K_Ca_ channel homologues are present in all protozoa except *T. vaginalis*. The K_Ca_2/3 channel homologues in many cases contain consensus CaMBDs [65], while the K_Ca_1 homologues show similarity to the discontinuous Ca^2+^-binding RCK domains of K_Ca_1 channels [60]–[63]. Searches of parasite genomes using the sequence of human CaM showed that genes encoding homologues of CaM are present in the genomes of all protozoa examined in this study, consistent with a previous report [109] except that we additionally identified a CaM homologue in *C. parvum* (*data not shown*). In addition, effects attributed to functionally expressed CaM have been reported in many pathogenic protozoa [110]–[117]. This suggests that CaM is a likely Ca^2+^-binding modulator of protozoan K_Ca_ channel homologues with consensus CaMBDs. RCK domains of K_Ca_ channels can directly confer regulation by Ca^2+^, Mg^2+^, Na^+^ or H^+^ ions [61]. The discontinuous nature of RCK domain structure [118] and the occurrence of ion binding sites at interfaces between RCK subunits [62], [63], [119], as well as between RCK subunits and other regions of the channel [120], makes prediction of the ionic specificity of RCK domains difficult on the basis of sequence alone. Experimental testing will be required to determine which of these potential modulatory factors acts at the various parasite K_Ca_1 homologues identified.

CNBD-containing K_CNG_ channels show great variability in their occurrence within genomes. They are very rare in prokaryotes [7], but are abundant in *Paramecium* [12], [13]. The relative abundance of CNBD-containing K_CNG_ homologues in *T. vaginalis* is consistent with a large number of genes encoding adenylyl cyclases in this organism [121]. This suggests that these proteins may play roles in diverse cyclic nucleotide signalling pathways in this parasite and that these pathways may be possible therapeutic targets.

K_ir_ channels are widespread and have diverse functions in many organisms [71]. Many different stimuli affect the activity of K_ir_ channels, including G-proteins, phospholipids, divalent and monovalent cations, as well as ATP [71]. Which stimuli affect the protozoan K_ir_ homologues identified here is difficult based on sequence alone, and will require future experimental testing.

Some parasite K^+^ channel subunit homologues had GYX-containing putative selectivity filters, suggesting that they may be non-selective or non-functional. Some of these proteins may be paralogues of K^+^ channels with canonical GYG-containing selectivity filters. Interestingly, GYX-containing K^+^ channel homologues exist in some prokaryotes, but their ionic selectivity and function are unknown [7]. In contrast, genes encoding GYX-containing K^+^ channel subunit homologues are not found in the human genome (*data not shown*). This suggests that if functional, the parasite GYX proteins (whether K^+^-selective channels or not) may constitute unique drug targets in protozoan parasites.

In addition to the K^+^ channel homologues identified, it is possible that other K^+^-permeable channels with novel architectures may exist in parasites. For example, a novel family of adenylyl cyclases found in members of the Alveolata subkingdom of protozoa, such as *Paramecium* and *P. falciparum*, may also possess K^+^ channel activity [122]–[124]. These proteins are involved in parasite exocytosis and infectivity [125]. These putative adenylyl cyclase/K^+^ channel fusion proteins have an architecture that is radically different from that of canonical K^+^ channels, with a GXG motif situated *after* TMD6 [124]. Only a single study has thus far indicated their possible intrinsic function as K^+^-conductive (but relatively non-selective) ion channels, using a member of this protein family from *Paramecium* [122]. Hence this family of proteins cannot at present be definitively categorized as functional K^+^ channel homologues in parasites, but may in future be shown to contribute to K^+^ flux in these organisms. Consistent with previous reports [123]–[125] our analyses identified genes encoding homologues of these fusion proteins in *Plasmodium* spp., *T. gondii* and *Cryptosporidium* spp. (legend to **Table 1**). Genes encoding homologues of these proteins were absent in the genomes of all other parasites examined.

Genes encoding homologues of K^+^ channel auxiliary subunits are absent in the genomes of the protozoa examined. This suggests that functional K^+^ channel complexes in these organisms may lack the diversity of human K^+^ channel complexes. In addition, since auxiliary subunits can dictate the pharmacology of native K^+^ channel complexes in mammalian cells [20], [92], [93], the pharmacology of native K^+^ channel complexes in parasites may differ substantially from those of humans. It is also possible that unique and as yet unidentified auxiliary subunits exist in protozoa, which are unrelated to currently known auxiliary subunits of K^+^ channels in other organisms.

### K^+^ channels in parasitic protozoa and their free-living relatives

In contrast to the relatively few genes encoding K^+^ channel subunit homologues in parasitic protozoa, the genome of their free-living ciliate relative *Paramecium tetraurelia* has several hundred genes encoding K^+^ channel subunits [13]. Whether this dramatic difference arose due to the acquisition of a parasitic existence in some protozoa is unclear.

BLAST searches using the sequences of KcsA as well as human K_v_1.2, K_ir_2.1 and K_Ca_1.1 proteins suggest that the genome of the free-living flagellate *Monosiga brevicollis* encodes at least nine distinct K^+^ channel subunit homologues (*data not shown*), compared with the smaller number in kinetoplastid parasites (*eg.* only two in *T. brucei*). Whether the fewer K^+^ channel homologues in the flagellate parasites examined here is due to a parasitic existence is unclear.

### Protozoan K^+^ channels as therapeutic targets

K^+^ channels are critical for cellular homeostasis and signal transduction, and pharmacological modulation of these channels can lead to marked changes in cell growth and viability [36], [77], [126], [127]. Disrupting K^+^ channel function in *P. falciparum* and *P. berghei* severely compromises survival of these parasites [16], [37], suggesting that parasite K^+^ channels may be attractive drug targets for treatment of parasitic disease. Interestingly, genetic disruption of K^+^ channel function affects different life cycle stages in *P. falciparum* and *P. berghei*. In *P. falciparum* the asexual blood stage is affected [15], while in *P. berghei* the sexual mosquito stage is severely affected but the asexual stage is relatively unaffected [16]. While the human genome encodes more than 70 K^+^ channel subunits [13], the genomes of pathogenic protozoa each contain only a small number of genes encoding homologues of K^+^ channel subunits (**Table 1**). This striking lack of redundancy in the K^+^ channel complement of protozoan parasites further suggests that these channels might be effective therapeutic drug targets.

Several drugs that inhibit K^+^ channel activity are known to be toxic to protozoan parasites. In many cases the primary anti-parasitic mode of action of these drugs is likely to be on processes other than K^+^ flux. However, K^+^ channel-blocking drugs may alter the activity of the protozoan parasite K^+^ channel homologues described in this study, perturb cellular K^+^ homeostasis and contribute to the decreased survival of parasites. For example, chloroquine blocks K_ir_ channels [128] and is also toxic to various species of *Plasmodium* [129]. The anti-trypanosomal drug pentamidine also blocks K_ir_ channels [130]. The K_ir_6 channel antagonist glibenclamide [131] and the K^+^ channel blocker amiodarone [132] are both known to be lethal to *Leishmania mexicana*. The K^+^ channel blocker amantadine is toxic to *P. falciparum* [133]. In addition, bicuculline and tubocurarine, which block some K^+^ channels in addition to other targets [134], [135] are also toxic to *P. falciparum* [37]. Functional evidence will be required before any causal links between inhibition of parasite K^+^ channel activity and anti-parasitic effects can be established.

Drugs that *activate* K^+^ channels may also be toxic to protozoan parasites. This has not been experimentally tested in protozoa, although it has been reported to play a role in the action of some drugs against parasitic nematodes [136]. Yeast also exhibit a loss of viability in response to a K^+^ channel-activating toxin which leads to excessive K^+^ flux [11]. Protozoan parasites within mammalian cells could be uniquely susceptible to drug-induced activation of parasite plasma membrane K^+^ channels, as the relatively high cytosolic K^+^ concentration of the host cell might allow K^+^ overload of the parasite, a scenario rarely encountered by mammalian cells due to the relatively low concentration of extracellular K^+^. Well-defined K^+^ channel openers exist which have minimal negative effects in humans, and indeed some of these are used as anti-nematode drugs [136], muscle relaxants, antiepileptics and analgesics [137], [138]. It is possible that the effectiveness of this strategy would be reduced in parasites that lower the cytosolic K^+^ concentration of the host cell, such as *Plasmodium* spp. [101]–[104].

The well-characterized pharmacology of K^+^ channels, together with sequence differences between human and parasite proteins, suggests that specific block of parasite K^+^ channel homologues might be an achievable target. For example, determinants of isoform-specific block of K_Ca_2 channels by drugs and toxins have been described in the outer pore region [78], [139], [140], where sequence differences exist between human and parasite homologues. This suggests avenues for development of drugs that specifically block parasite K_Ca_ channel homologues rather than human isoforms. Pharmacological blockers of other K^+^ channels also bind within the pore region and in some cases their binding affinity can be predicted with confidence from primary protein sequence alone. For example, TEA binds to the outer pore and a residue following the selectivity filter (GXGXX**X**) is a critical determinant of its binding affinity [75]. Channels with an aromatic residue at this position almost universally display a high-affinity interaction with TEA. Hence it is likely that the K^+^ channel homologues in *Plasmodium* spp. (XP_001348796, XP_001615733 and XP_001348796) as well as the homologue in *E. histolytica* (XP_655083), which all possess a phenylalanine or tyrosine residue at this locus, will display high affinity block by TEA. As discussed earlier, the lack of homologues of mammalian K^+^ channel auxiliary subunits in the protozoan parasites examined here also suggests a lack of diversity in native parasite K^+^ channel complexes and an opportunity for selective targeting of these parasite K^+^ channels by drugs.

This study presents the opportunity for cloning and functional characterization of K^+^ channels in pathogenic protozoa, and suggests that rational design of therapeutic strategies targeted against parasite K^+^ channels may be an attractive prospect. Future studies of parasite genomes and cellular signalling will lead to a deeper understanding of the presence and function of these channels in pathogenic parasites.

## Materials and Methods

### Genome analysis, sequence alignments and topology analysis

Analysis of genomes, sequence alignments and topology analysis were conducted as reported previously [2]. BLASTP and TBLASTN searches of protozoan genomes were carried out against the National Center for Biotechnology (NCBI) genomic protein databases. In multiple sequence alignments (ClustalW2) asterisks below the alignment indicate positions that have a single fully conserved residue, while colons below the alignment indicates positions that have residues with highly similar properties (scoring >0.5 in the Gonnet PAM 250 matrix, ClustalW2).

BLASTP analysis was carried out using the sequences of the following diverse human K^+^ channels (protein accession number in parentheses): K_v_1.2 (NP_004965.1), K_v_7.1 (NP_000209.2) and K_v_11.1 (hERG1) (Q12809.1); K_ir_1.1 (ROMK1) (NP_000211.1), K_ir_2.1 (IRK1) (NP_000882.1), K_ir_3.1 (GIRK1) (NP_002230.1), K_ir_4.1 (P78508.1), K_ir_5.1 (Q9NPI9.1), K_ir_6.1 (K_ATP_1) (Q15842.1), K_ir_6.2 (NP_000516.3) and K_ir_7.1 (CAA06878.1); K_2P_1.1 (TWIK1) (NP_002236.1), K_2P_2.1 (TREK1) (NP_001017425.2), K_2P_3.1 (TASK1) (NP_002237.1), K_2P_13.1 (THIK1) (NP_071337.2), K_2P_16.1 (TALK1) (NP_001128577.1) and K_2P_18.1 (TRESK2) (NP_862823.1); K_Ca_1.1 (BK) (NP_001154824.1), K_Ca_2.1 (SK1) (NP_002239.2), K_Ca_2.2 (SK2) (NP_067627), K_Ca_3.1 (IK/SK4) (NP_002241.1) and K_Ca_4.1 (SLACK/K_Na_) (NP_065873.2). Other K^+^ channel sequences were also used to search for parasite homologues, including: *Plasmodium falciparum* PfKch1 (XP_001350669.2) and PfKch2 (XP_001348796.2) [15], bacterial KcsA (P0A334), bacterial cyclic nucleotide-gated MlotiK1 (Q98GN8.1), archaeal depolarization-activated K_v_AP (Q9YDF8.1), archaeal hyperpolarization-activated MVP (Q57603.1), archaeal Ca^2+^-activated MthK (O27564.1), and fungal TOK1 (CAA89386.1). Plant K^+^ channel sequences were also used, including: the vacuolar outwardly rectifying, calcium-regulated vacuolar two-pore TPK1 channel (NP_200374.1); vacuolar KCO3 (NP_001190480.1); the pollen plasma membrane TPK4 (NP_171752.1), the inward rectifier KAT1 (NP_199436.1), the outward rectifier SKOR (pore region of NP_186934.1, residues 271–340 to avoid ankyrin hits), and AKT1 (NP_180233.1). Sequences of human CNGA1 (EAW93049; full-length, and TMD residues 200–420), and CNGB1 (NP_001288), as well as human HCN2 (NP_001185.3; full-length, and TMD residues 200–470) were also used to search for parasite homologues. In addition, the sequence of a novel putative adenylyl cyclase/K^+^ channel fusion protein in *P. falciparum* (PfAC1 or PfACα; XP_001348216) was used to search for homologues in other parasites. Sequences of K^+^ channel auxiliary subunits that were used to search for parasite homologues include: human KCNE1 (NP_001121142.1), human K_v_β1 (NP_751892.1), human KChIP1 (NP_001030009), human BKβ (NP_004128.1), human SUR1 (NP_000343) and human SUR2A (NP_005682). The sequences of both the isolated nucleotide-binding domains of SUR1 and SUR2A and the sequences outside these nucleotide-binding regions were also used to search for parasite homologues. Results of BLASTP analysis were confirmed using TBLASTN analysis in all cases. Default BLAST parameters for assessing statistical significance and for filtering were used in all cases (*ie.* an Expect threshold of 10, and SEG filtering).

Several procedures ensured that hits were probable K^+^ channel homologues. Firstly, the occurrence of multiple putative TMDs was confirmed using TOPCONS [141]. Secondly, reciprocal BLASTP searches (non-redundant protein database at NCBI) were undertaken, using identified parasite hits as bait, and only proteins that gave the original mammalian protein family as hits were analyzed further. Thirdly, conserved domains were identified using the Conserved Domains Database (NCBI). Lastly, only hits with regions of sequence similarity that encompassed the selectivity filter sequence of the K^+^ channel subunit used as bait were acknowledged. K_CNG_ channel parasite homologues were identified as proteins showing sequence similarity in both the pore *and* the CNBD regions. Where a hit showed similarity to more than one human K^+^ channel, the parasite protein was designated as a homologue of the human channel to which it showed greatest sequence similarity (ClustalW2) and which contained similar putative functional domains.

For phylogenetic analysis, multiple sequence alignments were constructed with MUSCLE v3.7 using default parameters. After use of GBLOCKS at low stringency to remove regions of low confidence, and removal of gaps, Maximum Likelihood analysis was undertaken using PhyML v3.0 (WAG substitution model; 4 substitution rate categories; default estimated gamma distribution parameters; default estimated proportions of invariable sites; 100 bootstrapped data sets). The phylogenetic tree is shown using TreeDyn (v198.3). MUSCLE, GBLOCKS, PhyML and TreeDyn are all functions of Phylogeny.fr (http://www.phylogeny.fr/) [142].

CaM-binding sites were identified using The Calmodulin Target Database search facility (http://calcium.uhnres.utoronto.ca/ctdb) [143]. Secondary structure of proteins was predicted using SABLE (http://sable.cchmc.org) [144].
